# Detection of luminescence of radicals from TiO_2_ plate during alpha particle irradiation

**DOI:** 10.1117/1.JBO.25.9.096008

**Published:** 2020-09-26

**Authors:** Seiichi Yamamoto

**Affiliations:** Nagoya University Graduate School of Medicine, Department of Integrated Health Science, Nagoya, Japan

**Keywords:** titanium dioxide, alpha particles, photodynamic therapy, luminescence, radical production, Cerenkov-light

## Abstract

**Significance:** A significant amount of radical related luminescence is observed from the titanium dioxide (${\mathrm{TiO}}_{2}$) plate during irradiation of alpha particles although alpha particles do not emit Cerenkov-light.

**Aim:** ${\mathrm{TiO}}_{2}$ is a promising material for application to photodynamic therapy in combination with positron radionuclides that emit Cerenkov light. However, it is not clear that radicals are produced by the irradiation of alpha particles, since although Cerenkov light is required to produce radicals, alpha particles do not produce Cerenkov light.

**Approach:** To clarify this point, the author irradiates alpha particles to a ${\mathrm{TiO}}_{2}$ plate and measured the produced luminescence of the plate, which indicates the radical production in ${\mathrm{TiO}}_{2}$.

**Results:** A significant amount of luminescence was observed from the ${\mathrm{TiO}}_{2}$ plate during irradiation of alpha particles. The spectrum of luminescence from the ${\mathrm{TiO}}_{2}$ plate during this irradiation was the same as that emitted by ultraviolet (UV) light irradiated luminescence, which actually showed radical production in a ${\mathrm{TiO}}_{2}$ plate. This luminescence was not attributed to the UV light from the air scintillation by alpha particles but to the direct irradiation of alpha particles to the ${\mathrm{TiO}}_{2}$ plate.

**Conclusions:** A significant amount of luminescence from ${\mathrm{TiO}}_{2}$ plate was detected during irradiation of alpha particles. The luminescence is thought to be emitted from the radicals produced by the direct alpha particle irradiation to the ${\mathrm{TiO}}_{2}$ plate.

## 1 Introduction

Titanium dioxide (${\mathrm{TiO}}_{2}$) is an attractive and promising material for its applications to environmental fields with its photocatalysis characteristics via irradiation of ultraviolet (UV) light.^1^^–^^10^ Furthermore, in biomedical research fields, ${\mathrm{TiO}}_{2}$ powder offers a promising sensitizer for therapies in combination with UV light^11^ and ultrasound irradiation.^12^^,^^13^ A photodynamic therapy using ${\mathrm{TiO}}_{2}$ nanoparticles combined with Cerenkov-light irradiation from positrons was also reported.^14^^,^^15^ In these works of photodynamic therapy, positron emitters and ${\mathrm{TiO}}_{2}$ nanoparticles are injected into mice with tumors, and the mice injected with both of these showed longer survival times. Here, the mechanism of therapy was explained as follows. UV light in Cerenkov light from positrons produced radicals in the tumor of a mouse where ${\mathrm{TiO}}_{2}$ nanoparticles had accumulated, and the tumor was killed by the radicals. However, this explanation has faced the criticism that the intensity of Cerenkov light from positrons is too small to produce enough radicals from the ${\mathrm{TiO}}_{2}$ particles for therapy. The low intensity of Cerenkov light produces a small number of radicals from ${\mathrm{TiO}}_{2}$, thus it is difficult for this viewpoint to explain the effectiveness of positron-based photodynamic therapy.^16^ Consequently, another mechanism of producing radicals is needed to explain the effectiveness of these photodynamic therapies using radionuclides.

**Fig. 1:**
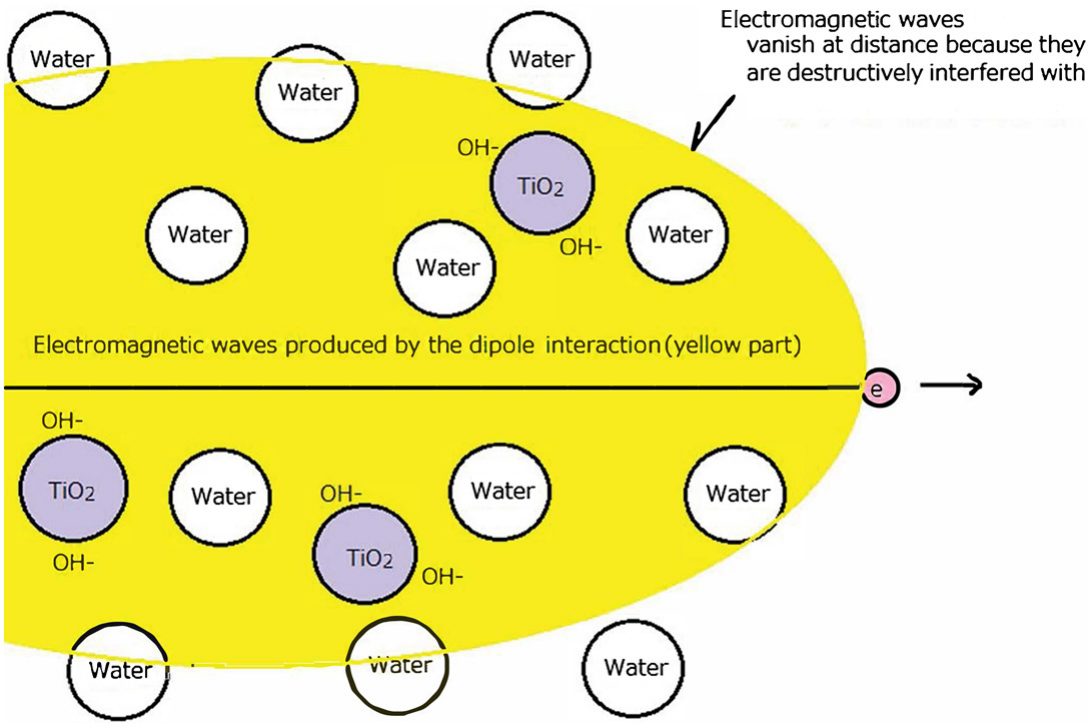
Conceptual illustration of producing radicals by electromagnetic waves (luminescence of water at lower energy than Cerenkov-light threshold) and ${\mathrm{TiO}}_{2}$.

Recently, the author found luminescence of water at a lower energy than the Cerenkov-light threshold.^17^^–^^20^ The luminescence had a similar light spectrum to that of Cerenkov light and is thought to be produced by the same mechanism as Cerenkov light: electromagnetic waves produced by the dipole interaction of moving electrons with water molecules at lower energy than the Cerenkov-light threshold.^21^ Although the intensity of the luminescence of water is low from a distance, the original electromagnetic wave intensity produced by the dipole interaction of moving electrons with water molecules at the produced points is thought to be much higher than the observed light as schematically shown in Fig. 1. However, the intensity of the electromagnetic waves (light) are reduced at a distant point because the electromagnetic waves are incoherent and emitted almost simultaneously, thus the electromagnetic waves interfere with each other destructively and almost no electromagnetic waves are observed from a distance.^22^^,^^23^ The luminescence of water found by the author was the small amount of remaining electromagnetic waves that were believed to be invisible from a distance before the author discovered the light.^21^^–^^23^

The author realized that the intensity of the produced electromagnetic waves (original light of luminescence lower than Cerenkov light threshold) before vanishing must be very high, much higher than Cerenkov light. This is attributed to Cerenkov light being produced from only a small fraction of the original produced light.^22^^,^^23^ Because the moving electron produces the electromagnetic waves by interaction with electrons in molecules, if ${\mathrm{TiO}}_{2}$ particles exist near the electron as shown in Fig. 1, the electromagnetic waves reacting with ${\mathrm{TiO}}_{2}$ particles produce many radicals before vanishing. To clarify whether the proposed mechanism actually occurred, the author directly irradiated alpha particles on a ${\mathrm{TiO}}_{2}$ plate and measured the produced radicals in the plate using a high-sensitivity cooled charge-coupled device (CCD) camera.

## 2 Materials and Methods

### 2.1 Principle of Detecting Radicals in TiO_2_ During Irradiation of Alpha Particles

It is well established that luminescence at a wavelength between 400 and 600 nm emitted from ${\mathrm{TiO}}_{2}$ during irradiation of UV light reflects the quantity of produced radicals.^24^^–^^28^ Therefore, the author irradiated alpha particles on a ${\mathrm{TiO}}_{2}$ plate and measured the luminescence from the plate using a high-sensitivity cooled CCD camera. The author also measured the spectrum of the produced light from the ${\mathrm{TiO}}_{2}$ plate to confirm that the light is actually from the radicals produced in ${\mathrm{TiO}}_{2}$. The intensity of the light emitted during irradiation of the alpha particles was compared with that during irradiation of UV light from the air scintillation of alpha particles, which had almost the same intensity level as Cerenkov light.

### 2.2 Experimental Set-Up

**Fig. 2:**
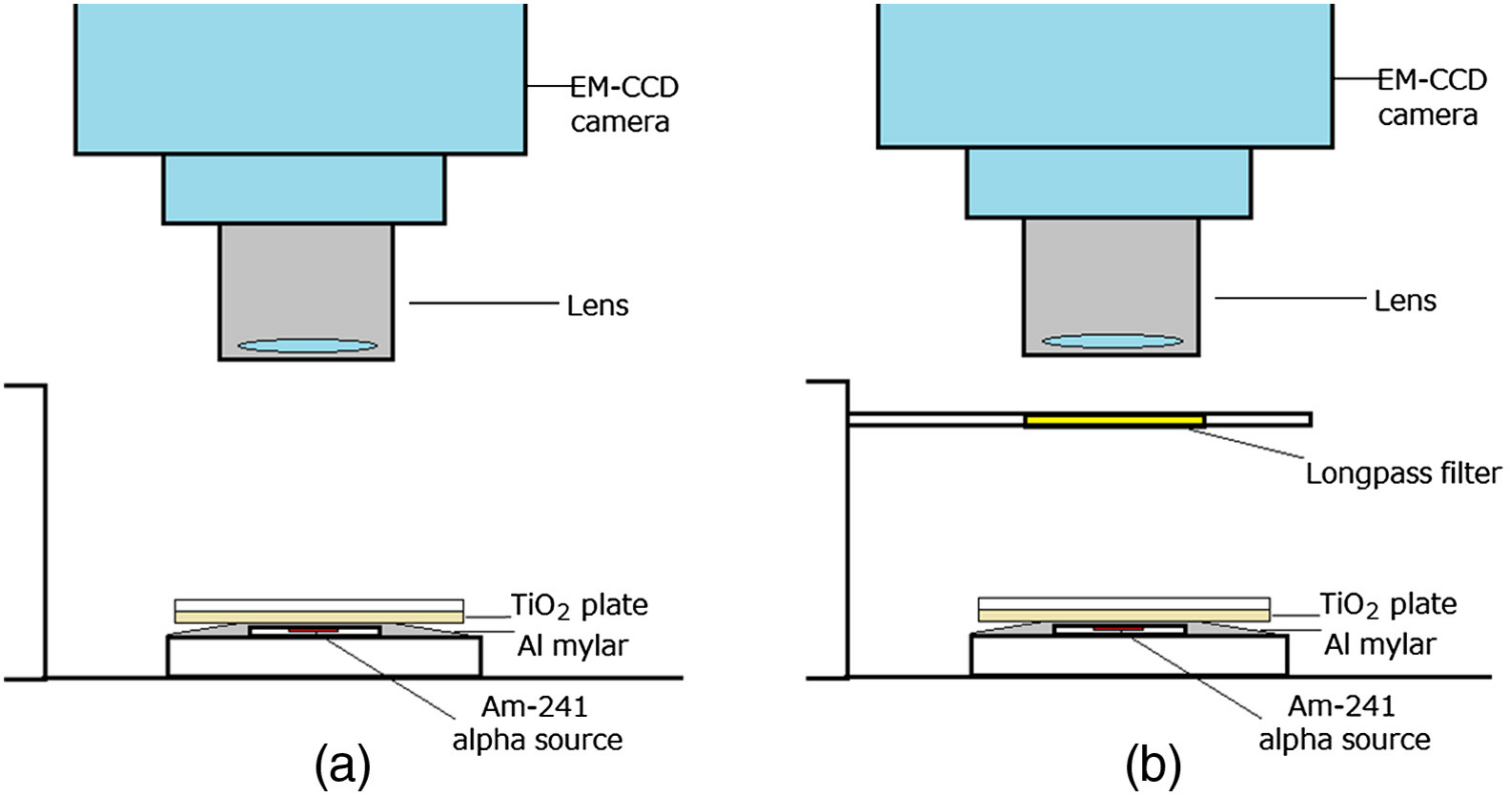
Set up during measuring produced radical distributions of ${\mathrm{TiO}}_{2}$ plate during irradiation of alpha particles: (a) without and (b) with optical filter.

Figure 2 shows the measurement set up for produced radical intensity and distributions of the ${\mathrm{TiO}}_{2}$ plate during irradiation of alpha particles. On the americium-241 (Am-241) alpha source covered by an aluminized Mylar film, a plate pasted with ${\mathrm{TiO}}_{2}$ powder was set. For measuring the light from the ${\mathrm{TiO}}_{2}$ plate, the image of the ${\mathrm{TiO}}_{2}$ plate during irradiation of alpha particles was acquired by an electron-multiplied cooled CCD (EM-CCD) camera from above as shown in Fig. 2(a). When measuring the spectrum of the light from ${\mathrm{TiO}}_{2}$ or the scintillation of air, one of the long-pass filters was set in front of the lens of the EM-CCD camera, which was used to measure the images as shown in Fig. 2(b).

**Fig. 3:**
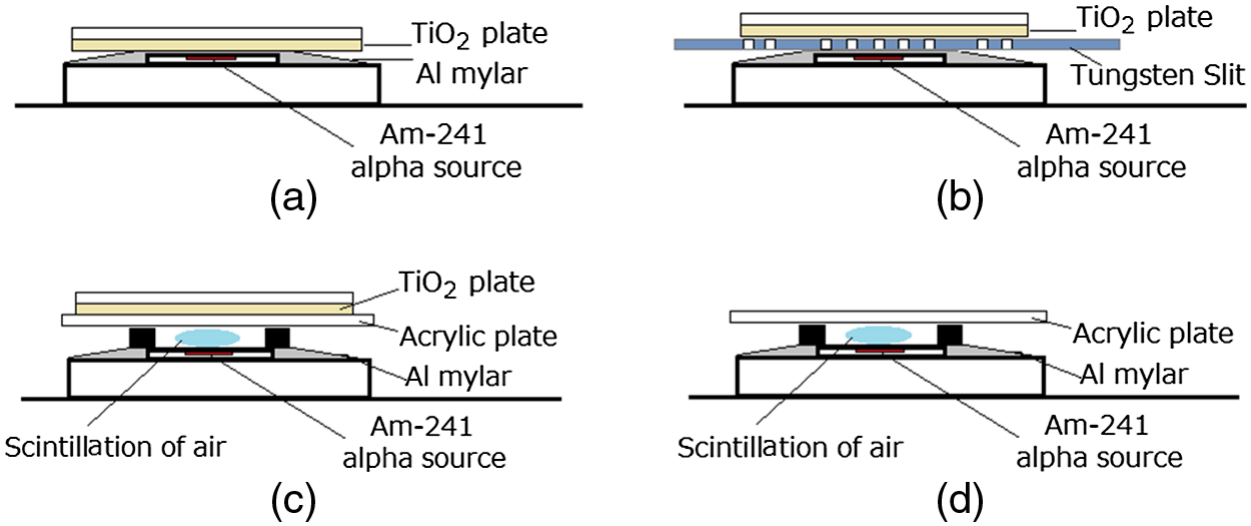
Set up to measure luminescence images of ${\mathrm{TiO}}_{2}$ plate under various conditions: (a) standard imaging of luminescence of ${\mathrm{TiO}}_{2}$ plate during irradiation of alpha particles, (b) imaging of luminescence of ${\mathrm{TiO}}_{2}$ plate with tungsten plate during irradiation of alpha particles, (c) imaging of luminescence of ${\mathrm{TiO}}_{2}$ plate during irradiation of scintillation of air, and (d) imaging of scintillation of air.

The author measured the luminescence of the ${\mathrm{TiO}}_{2}$ plate under various conditions. To measure the images of the ${\mathrm{TiO}}_{2}$ plate during irradiation of alpha particles, the plate was set directly on the Am-241 alpha source as shown in Fig. 3(a). In this case, there is no space between the Am-241 alpha source and the ${\mathrm{TiO}}_{2}$ plate and the luminescence of the ${\mathrm{TiO}}_{2}$ plate during irradiation of alpha particles was measured.

To check that the position of produced luminescence was the same as the position of the irradiated alpha particles, a thin tungsten plate was set between the Am-241 alpha source and the ${\mathrm{TiO}}_{2}$ plate as shown in Fig. 3(b). In this case, there was also no space between the Am-241 alpha source and the ${\mathrm{TiO}}_{2}$ plate, and the luminescence image of the ${\mathrm{TiO}}_{2}$ plate was measured.

To measure the images of the ${\mathrm{TiO}}_{2}$ plate by irradiation of UV light from the air scintillation produced by alpha particles, the plate was set on a UV transparent acrylic plate, and the UV light from air scintillation produced by the alpha particles was irradiated to the ${\mathrm{TiO}}_{2}$ plate as shown in Fig. 3(c). In this case, there was a 2-mm space between the Am-241 alpha source and the UV transparent acrylic plate to produce the air scintillation by alpha particles in this space. The alpha particles were absorbed by the acrylic plate and were not detected by the ${\mathrm{TiO}}_{2}$ plate. To measure the scintillation of air in the space during irradiation of alpha particles, the ${\mathrm{TiO}}_{2}$ plate was removed and imaging of the air scintillation was carried out as shown in Fig. 3(d). The imaging of the UV transparent acrylic plate during irradiation of alpha particles was also conducted to check whether the light production by the acrylic plate during irradiation of alpha particles was negligible.

The author also measured the image of a plastic scintillator (NE-102A) during irradiation of alpha particles to quantify the measured intensity of the images assuming the light emission of plastic scintillator for alpha particles is 1000  photons/MeV.

### 2.3 TiO_2_ Plate and Alpha Source

**Fig. 4:**
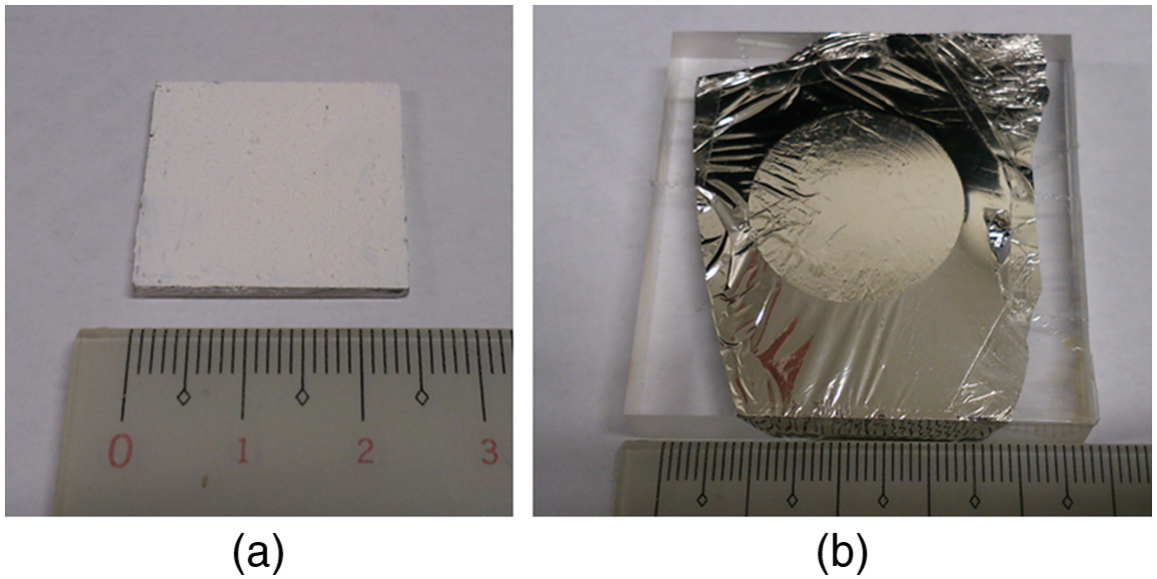
Photo of fabricated (a) ${\mathrm{TiO}}_{2}$ plate and (b) alpha source used for imaging experiments.

${\mathrm{TiO}}_{2}$ powder (anatase, average diameter 100 to 150 nm, Nacalai Tesque, Kyoto, Japan) was used for fabricating the ${\mathrm{TiO}}_{2}$ plate. ${\mathrm{TiO}}_{2}$ powder was mixed with a binder (polyvinyl alcohol) at a weight ratio of 1:1 and made a uniformly mixed ${\mathrm{TiO}}_{2}$ solution. On an acrylic plate (25  mm×25  mm×1.5  mm thick), this ${\mathrm{TiO}}_{2}$ solution was pasted with a thickness of ∼60  μm [Fig. 4(a)]. This was thick enough to absorb all the energy of the alpha particles in ${\mathrm{TiO}}_{2}$. The imaging of the binder during irradiation of alpha particles was also conducted to check whether the light production of the binder during irradiation of alpha particles was negligible.

The alpha source was Am-241 (5.5 MeV) with an activity of 2-MBq, and the source’s active area was 10  mm×10  mm. To avoid scintillation of the dust on the source, the active area was covered with an aluminized Mylar film to absorb the light on the source surface [Fig. 4(b)].

The refractive index of ${\mathrm{TiO}}_{2}$ is 2.52, so Cherenkov light threshold energy is low (46 keV for 2.52). The Cherenkov light threshold is lower than 59 keV, which is the energy of the gamma photons from Am-241 and it is possible to emit Cherenkov light by 59-keV gamma photons. The author checked the intensity of the ${\mathrm{TiO}}_{2}$ plate during irradiation of 59-keV gamma photons from Am-241 by inserting a black paper between the Am-241 source and ${\mathrm{TiO}}_{2}$ plate to absorb alpha particles and compared the intensities with that of alpha particles.

### 2.4 Imaging System

**Fig. 5:**
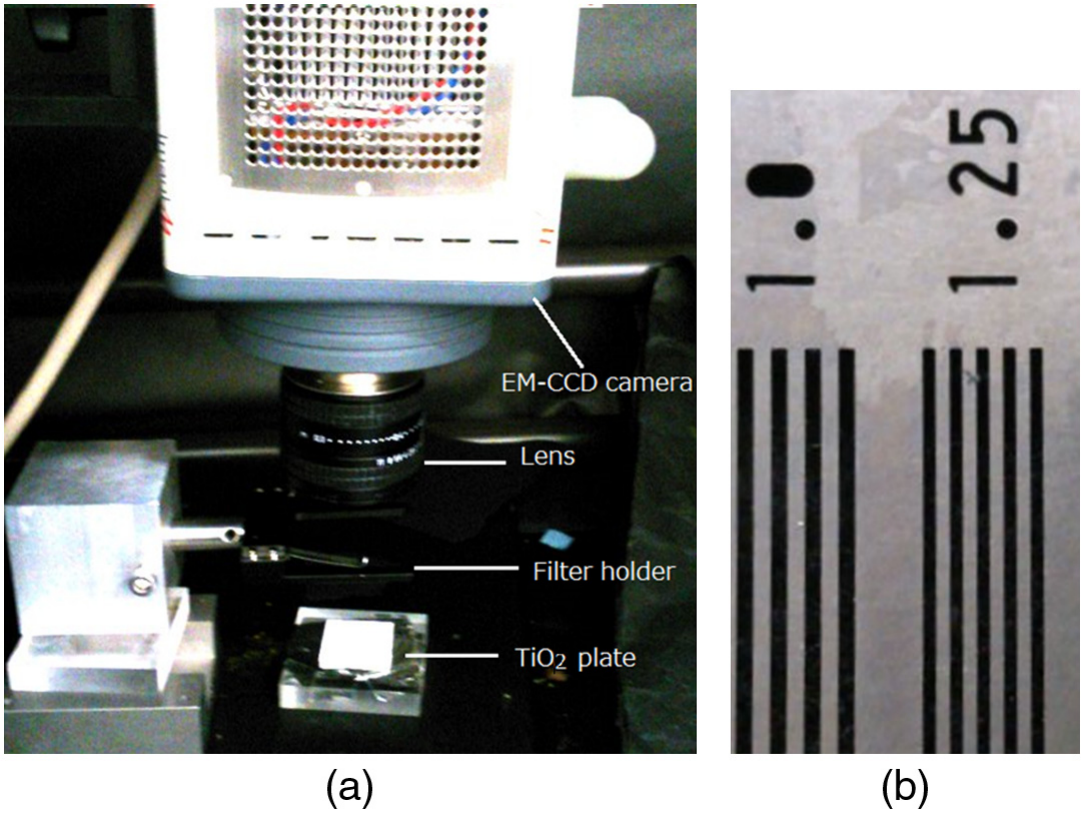
(a) Photo of imaging system to measure luminescence of ${\mathrm{TiO}}_{2}$ plate and (b) photo of tungsten slits.

The imaging system used to measure the luminescence of a ${\mathrm{TiO}}_{2}$ plate is shown in Fig. 5(a). The system used for the imaging was a cooled EM-CCD camera (ImagEM, Hamamatsu Photonics, Japan) operating at −65°C with a C-mount F-0.95 lens set ∼8  cm from the source surface. An extension ring was used between the lens and the camera. Acquisition was conducted with 2×2 binning at a matrix size of 256×256. Each image was acquired for 10 s. The system was set in a dark box to shut out environmental light. The tungsten slits used for the imaging are shown in Fig. 5(b). The slit widths were 0.5 mm (1.0  line pair/mm) and 0.4 mm (1.25  line pair/mm), and the thickness was ∼0.1  mm.

The optical filters used were long-pass types longer than 350 nm (Asahi Spectra, LU0350), 450 nm (Asahi Spectra, LV0450), 550 nm (Asahi Spectra, LV0550), 650 nm (Asahi Spectra, LV0650), and 750 nm (Asahi Spectra, LI0750). The subtracted images were calculated from these images to produce bandpass images of 350 to 450 nm, 450 to 550 nm, 550 to 650 nm, and 650 to 750 nm. The intensities of these images were corrected for sensitivity distribution of the CCD camera and transmission of the lens and the light spectra were derived.

### 2.5 Image Processing

The acquired images were processed using public domain software (ImageJ). Noise spots due to the direct absorption of gamma photons by the CCD image sensor were eliminated using high-intensity and small-pixel information. A blank image was measured without light at the same acquisition time and then subtracted from each luminescence image for the correction of the offset value of the luminescence image. After setting a region of interest (ROI) on the luminescence part of each image, the average pixel value was calculated. The size of the ROI was ∼7  mm×7  mm.

## 3 Results

**Fig. 6:**
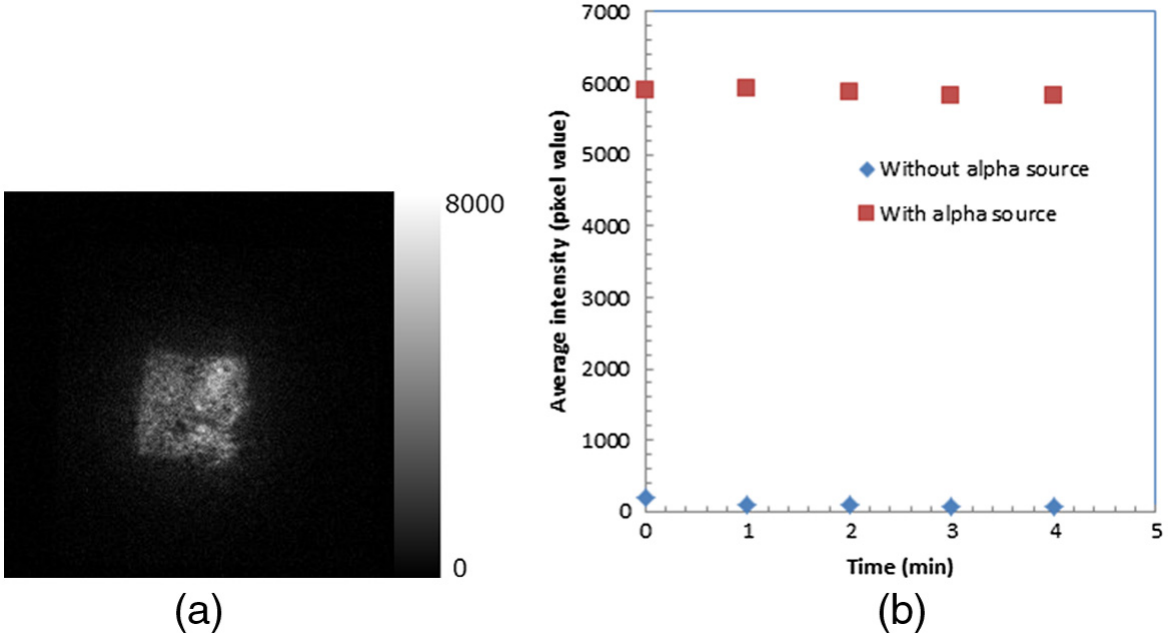
(a) Luminescence image of ${\mathrm{TiO}}_{2}$ plate during irradiation of alpha particles and (b) luminescence intensities with and without alpha source as a function of elapsed time after closing the dark box.

One of the luminescence images of the ${\mathrm{TiO}}_{2}$ plate during irradiation of alpha particles is shown in Fig. 6(a). The position where the alpha source directly contacts the ${\mathrm{TiO}}_{2}$ plate was imaged. The nonuniform distribution of the luminescence was due to the ${\mathrm{TiO}}_{2}$ plate’s nonuniformity. The luminescence intensities with and without an alpha source as a function of elapsed time after closing the dark box is shown in Fig. 6(b). In the image of the first frame, the background intensity was observed without a source at a level of 3% of that with a source. However, the background intensity of the ${\mathrm{TiO}}_{2}$ plate decreased to ∼1% of that with the alpha source.

**Fig. 7:**
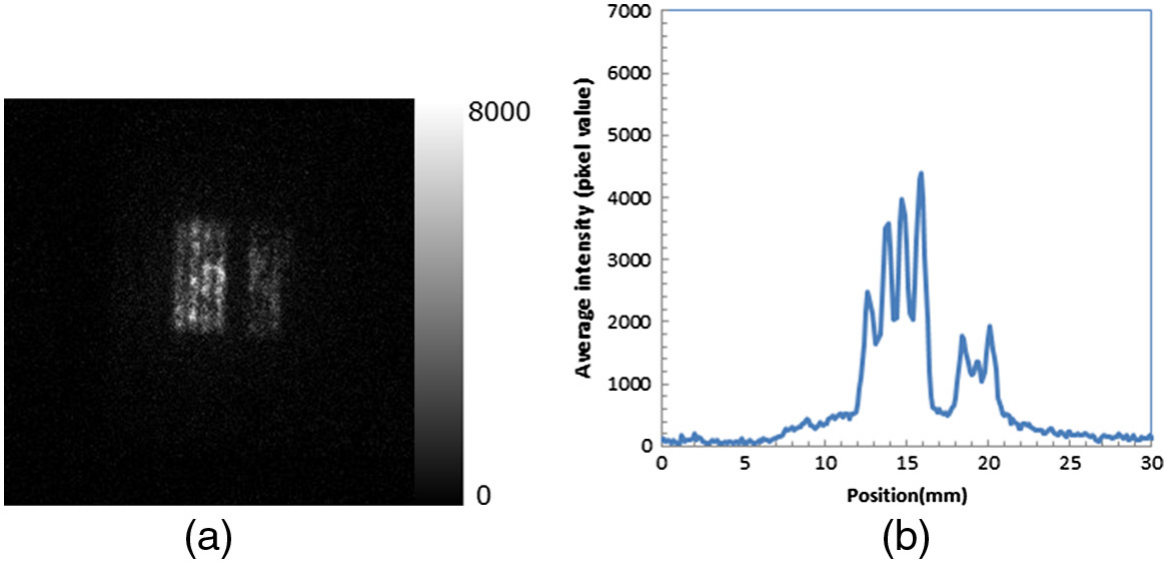
(a) Luminescence image of ${\mathrm{TiO}}_{2}$ plate with tungsten slit during irradiation of alpha particles and (b) profile of image in the horizontal direction.

A luminescence image of the ${\mathrm{TiO}}_{2}$ plate with a tungsten slit during irradiation of alpha particles is shown in Fig. 7(a). The profile of the image in the horizontal direction is shown in Fig. 7(b). The slit shape was clearly observed, and the 0.5-mm slits (left part of profile) were clearly resolved. The luminescence of the binder (polyvinyl alcohol) during irradiation of alpha particles was negligible (∼4% of the luminescence of ${\mathrm{TiO}}_{2}$). The intensity of the ${\mathrm{TiO}}_{2}$ plate during irradiation of the gamma photons (59 keV) from Am-241 was 1.5% of that during irradiation of alpha particles and it was also negligible.

**Fig. 8:**
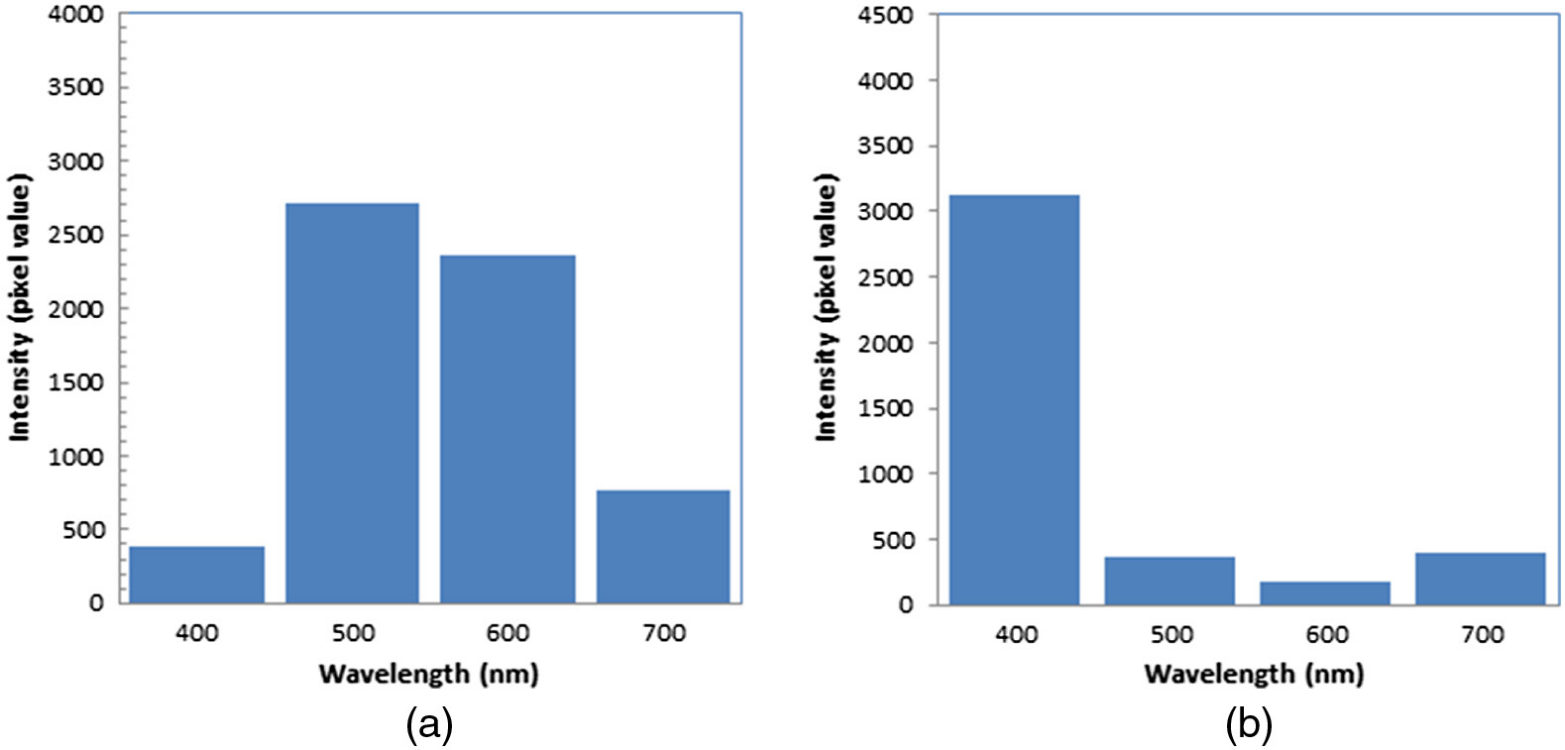
Light spectra of ${\mathrm{TiO}}_{2}$ luminescence during irradiation of (a) alpha particles and (b) scintillation of air.

Figure 8(a) shows the light spectra of ${\mathrm{TiO}}_{2}$ luminescence during the irradiation of alpha particles. The spectrum shows a peak around 500 nm, which was the same as the UV-irradiated spectrum of ${\mathrm{TiO}}_{2}$,^24^^–^^28^ indicating that the produced luminescence was the radicals produced in the ${\mathrm{TiO}}_{2}$ plate.

Figure 8(b) shows the light spectra of scintillation of air during irradiation of alpha particles to air. The spectrum shows a peak around 400 nm, which was the same as the light spectrum of air scintillation during alpha particle irradiations.^29^ Since the scintillation of air contained UV light shorter than 360 nm,^29^ it could be used for UV light to irradiate the ${\mathrm{TiO}}_{2}$ plate.

**Fig. 9:**
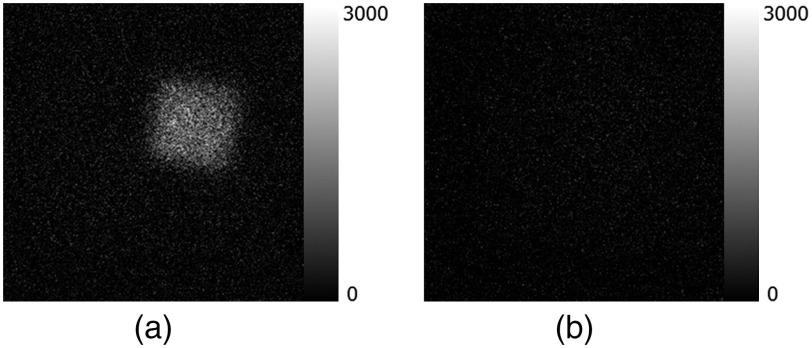
(a) Scintillation of air during irradiation of alpha particles and (b) luminescence of ${\mathrm{TiO}}_{2}$ plate during irradiation of scintillation of air.

Figure 9(a) shows the scintillation of air during irradiation of alpha particles. We could observe the blurred image of the scintillation of air. Figure 9(b) shows the luminescence of the ${\mathrm{TiO}}_{2}$ plate during irradiation of scintillation of air. The luminescence of the ${\mathrm{TiO}}_{2}$ plate during irradiation of scintillation of air was nearly invisible as shown in Fig. 9(b).

Table 1 summarizes the average intensities of luminescence of the ${\mathrm{TiO}}_{2}$ plate during irradiation of alpha particles and scintillation of air. The intensity of the luminescence of the ${\mathrm{TiO}}_{2}$ plate during irradiation of scintillation of air was only 1.6% of that during irradiation of alpha particles. The luminescence of the acyclic plate during irradiation of alpha particles was negligible (∼4% of the luminescence of ${\mathrm{TiO}}_{2}$).

**Table 1:** Average intensities of luminescence of ${\mathrm{TiO}}_{2}$ plate during irradiation of alpha particles and scintillation of air.

	Scintillation of air	${\mathrm{TiO}}_{2}$ plate during irradiation of alpha particles	${\mathrm{TiO}}_{2}$ plate during irradiation of scintillation of air
Average intensity	1050	5822	96
Ratio (alpha irradiation: 100%)	18	100	1.6
Photons/MeV	11	59	0.9
Photons/5.5 MeV alpha particle	61	326	5

## 4 Discussion

We measured the luminescence of a ${\mathrm{TiO}}_{2}$ plate during irradiation of alpha particles. The spectrum shown in Fig. 8(a) indicated that the luminescence of the ${\mathrm{TiO}}_{2}$ plate was attributed to the produced radicals in the ${\mathrm{TiO}}_{2}$ plate. The extremely small luminescence of the ${\mathrm{TiO}}_{2}$ plate during irradiation of the scintillation of air supports the notion that the luminescence of the ${\mathrm{TiO}}_{2}$ plate was not produced by the scintillation of air but by the direct irradiation of alpha particles to ${\mathrm{TiO}}_{2}$. One possible explanation is that the electromagnetic waves were produced by the dipole interaction of moving electrons with ${\mathrm{TiO}}_{2}$ or binder molecules; that was the origin of the luminescence of water and Cerenkov light.^21^^–^^23^ The electromagnetic waves are thought to vanish due to destructive interference with each other at a very close distance to the electron trajectory, but a certain distance is still needed for the waves to interfere with each other before vanishing. If the waves need one wavelength of the light to interfere with each other, the electromagnetic waves remain within ∼500  nm from the electron trajectory. Within that area, ${\mathrm{TiO}}_{2}$ particles produce radicals by the irradiation of the UV light in the electromagnetic waves.

The intensity of the scintillation of air during irradiation of alpha particles is ∼15  photons/MeV (Ref. 19) and the measured scintillation of air was 11  photons/MeV listed in Table 1, which is comparable intensity to that of Cerenkov light (∼20  photons) from positrons.^30^^,^^31^ When a similar intensity to Cerenkov light was irradiated to the ${\mathrm{TiO}}_{2}$ plate using the scintillation of air, almost no luminescence from radicals was observed in the ${\mathrm{TiO}}_{2}$ plate. This suggests that the radical production by positrons as previously reported^14^ was not from the Cerenkov light but might be attributed to the electromagnetic waves produced by the dipole interaction of moving positrons interacting with ${\mathrm{TiO}}_{2}$.

The luminescence intensities after closing the dark box were stable with and without an alpha source as shown in Fig. 6(b), indicating that the luminescence of the ${\mathrm{TiO}}_{2}$ plate was not from the phosphorescence by the irradiation of room light before measurement. There was very small phosphorescence (3% of the luminescence by the irradiation of alpha particles) observed in the ${\mathrm{TiO}}_{2}$ plate, but it still decreased within 1 min. Furthermore, it became obvious that the radical production in the ${\mathrm{TiO}}_{2}$ plate was stable during irradiation of alpha particles because the intensity of the luminescence was the same as shown in Fig. 6(b).

The luminescence image of the ${\mathrm{TiO}}_{2}$ plate with a tungsten slit during irradiation of alpha particles (Fig. 7) suggests that the radicals in the ${\mathrm{TiO}}_{2}$ plate were generally produced only in the areas where alpha particles were directly irradiated. This is good for photodynamic therapy because radicals are produced only in the areas where alpha particles are irradiated, not in other areas.

From the results of these measurements, radicals can be produced by other types of radiations such as those of beta particles, gamma photons, and x-rays. In the proposed mechanism, there is not a threshold energy of radiations to produce radicals. The radical production by irradiation of x-rays to ${\mathrm{TiO}}_{2}$ was previously reported.^32^ The author thinks that the radical production by irradiation of x-rays to ${\mathrm{TiO}}_{2}$ was attributed to the same mechanism as that of alpha particle irradiation occurring in ${\mathrm{TiO}}_{2}$.

## 5 Conclusion

A significant amount of luminescence from a ${\mathrm{TiO}}_{2}$ plate was observed during irradiation of alpha particles. The spectrum of the luminescence confirmed that this was from the radicals produced in ${\mathrm{TiO}}_{2}$. The luminescence was produced by the direct alpha particle irradiation to the ${\mathrm{TiO}}_{2}$ plate. With these results, alpha particles emitting radionuclides may have a potential for use in photodynamic therapy.
